# Harnessing stem cell and lineage reprogramming technology to treat cardiac fibrosis

**DOI:** 10.1186/s13619-023-00182-7

**Published:** 2023-12-11

**Authors:** Ni Zeng, Wei Tang, Yanghong Wu, Hang Fan, Shuanglun Xie, Nan Cao

**Affiliations:** 1 Advanced Medical Technology Center, The First Affiliated Hospital, Zhongshan School of Medicine, Sun Yat-Sen University, Guangdong, 510080 China; 2 NHC Key Laboratory of Assisted Circulation (Sun Yat-Sen University), Guangdong, 510080 China; 3 Key Laboratory for Stem Cells and Tissue Engineering (Sun Yat-Sen University), Ministry of Education, Guangdong, 510080 China; 4 Department of Cardiology, Sun Yat-Sen Memorial Hospital, Sun Yat-Sen University, Guangdong, 510120 China

**Keywords:** Cardiac fibrosis, Stem cell, Disease modelling, Drug screening, Transplantation, Reprogramming

## Abstract

Cardiac fibrosis is a pathological response characterized by excessive deposition of fibrous connective tissue within the heart. It typically occurs following cardiac injuries or diseases. However, the lack of suitable models for disease modeling and high-throughput drug discovery has hindered the establishment of an effective treatments for cardiac fibrosis. The emergence and rapid progress of stem-cell and lineage reprogramming technology offer an unprecedented opportunity to develop an improved humanized and patient-specific model for studying cardiac fibrosis, providing a platform for screening potential drugs and synchronously elucidating the underlying molecular mechanisms. Furthermore, reprogramming cardiac fibroblasts into cardiomyocyte-like cells to reduce scar volume and induce myocardial tissue regeneration is a promising approach in treating cardiac fibrosis. In this review, we summarize the current advancements in stem cell technologies applied to study cardiac fibrosis and provide insights for future investigations into its mechanisms, drug discovery as well as therapy method.

## Background

**Fig. 1:**
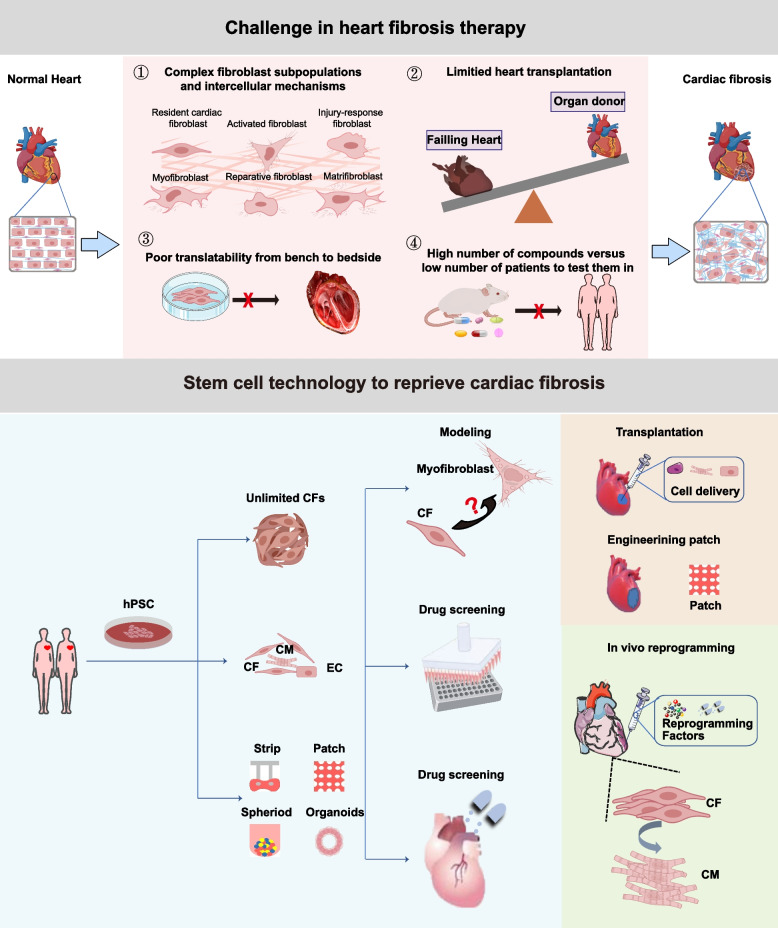
Challenges in treating cardiac fibrosis and potential solutions with stem cell technology. The red boxes above delineate some of the primary challenges encountered in the development of antifibrotic drugs, as discussed in this review. The subsequent blue box elucidates the applications of stem cell technology, wherein abundant cardiac cells derived from human pluripotent stem cells can be cultured into diverse structures to meet specific demands. Moreover, both the transplantation of products derived from human pluripotent stem cells and the in vivo reprogramming of cardiac fibroblasts into cardiomyocytes demonstrate promising potential for cardiac fibrosis therapy, as depicted by the adjacent brown and green boxes. TGF-β, transforming growth factor-β; Wnt/β-catenin, Wnt family/β-catenin; NF-κB, nuclear factor kappa B subunit; CF, cardiac fibroblasts; CM, cardiomyocytes; hPSC, human pluripotent stem cell; EC, endothelial cells

Cardiac fibrosis is a prevalent pathological alteration observed in the advanced stages of most cardiovascular diseases (CVDs). This debilitating condition is frequently associated with various cardiac disorders, resulting in impaired heart function and potentially life-threatening complications such as heart failure (Heidenreich et al. 2022). Pathological cardiac fibrosis is characterized by an excessive accumulation of fibrous tissue in the cardiac muscle, resulting from an uncontrolled tissue repair process primarily orchestrated by myofibroblasts. Myofibroblasts differentiating from fibroblasts upon stimulation are distinguished by the presence of smooth muscle actin (α-SMA) and increased production of extracellular matrix (ECM) proteins (Frangogiannis 2021; Ivey and Tallquist 2016; Ma et al. 2018). The process of myocardial fibrosis is associated with mechanical stimulation, paracrine effects among different cells, and the presence of pro-fibrotic factors or molecules derived from the circulatory system. Ultimately, this leads to an increased ratio of cardiac fibroblasts to cardiomyocytes (Fig. 1). The progressive accumulation of ECM replaces functional muscle tissues, resulting in adverse cardiac remodeling and significantly impairing myocardial contractile function.

Cardiac fibrosis can be attributed to non-infarcted injuries, including pressure overload, volume overload, metabolic dysfunction, and aging (Biernacka and Frangogiannis 2011; Borer et al. 2002). The activation of resident interstitial cell populations and other cell types such as cardiomyocytes and macrophages primarily drive the proliferation of activated myofibroblasts after myocardial injury (Brown et al. 2005; Frangogiannis 2012; Fu et al. 2018; Hinz 2010). The induction mechanism of fibrosis signals depends on the type of primary myocardial injury. The neurohumoral pathway activation directly stimulates fibroblasts or influences the immune cell population to activate them (Kurisu et al. 2003). Cytokines and growth factors, such as tumor necrosis factor (TNF)-α, Interleukin (IL) -1, IL-10, IL-11, transforming growth factor β (TGF-β) family members, and platelet-derived growth factor (PDGF), are secreted in the cardiac interstitium and specifically activating aspects of the fibrotic response (Flevaris et al. 2017; Gallini et al. 2016; Hofmann et al. 2014; Koitabashi et al. 2011). The secreted fibrotic mediators and matrix proteins bind to the cell surface receptors of fibroblasts, such as cytokine receptors, integrins, syndecans, transducing intracellular signaling cascades to regulate genes involved in ECM synthesis, processing, and metabolism (Berk et al. 2007; Frangogiannis 2017). The endogenous pathways involved in the negative regulation of fibrosis can protect the myocardium from an excessive fibrotic response which are crucial for heart repair. However, persistent heart damage disrupts the balance between fibrotic repair and its negative feedback regulation, leading to over-activation of myofibroblasts and excessive accumulation of ECM. Currently, heart fibrosis therapy faces several challenges (Fig. 1). The clinical treatments for cardiac fibrosis, including surgical interventions and pharmacotherapy, demonstrate limited efficacy or present insurmountable drawbacks. Surgical treatments for the prevention or management of advanced cardiac fibrosis encompass valve repair and heart transplantation. However, significant concerns including high surgical mortality rates, postoperative infections, and prolonged recovery periods cannot be disregarded (Marrouche et al. 2022). Moreover, the scarcity of heart donors and post-transplant immune rejection leave patients with cardiac fibrosis with few viable options.

Current pharmacological treatments for cardiac fibrosis primarily involve general anti-fibrotic drugs and cardiovascular protective medications (Spinale 2007). Pharmacologic therapies, such as angiotensin-converting-enzyme inhibitors, statins, aldosterone antagonists, and emerging therapies like histone deacetylase inhibitors, have been demonstrated to promote ‘reverse remodeling’. This has been proven to ameliorate cardiac fibrosis and subsequently reduce the burden of ventricular arrhythmia (Dimas et al. 2011; Massare et al. 2010) as well as the incidence of sudden cardiac death (Spinale 2007). However, despite anti-fibrotic drugs can partially inhibit the further expansion of fibrous tissue, their effectiveness in reversing established fibrosis is minimal (Zhao et al. 2022). Cardiovascular protective medications, such as beta receptor blockers and renin–angiotensin–aldosterone system (RAAS) inhibitors, can enhance cardiac function. However, they are unable to reverse existing fibrosis. Moreover, long-term medication usage may bring about drug tolerance and adverse effects (Rios et al. 2020). Currently, numerous small molecules or compounds are undergoing clinical trials for fibrosis treatment. However, most of them primarily focus on idiopathic pulmonary fibrosis, non-alcoholic steatohepatitis, or myelofibrosis (Zhao et al. 2022). Considering the presence of organ heterogeneity, the developments of specific drugs targeting myocardial fibrosis are significantly limited.

Obtaining human autologous heart cells poses a significant challenge (Smits et al. 2009), thus the utilization of animal models such as mice and rats is commonly favored in cardiac fibrosis research (Savoji et al. 2019). However, notable disparities exist between the physiological characteristics of human and mouse hearts, and animal models are unsuitable for high-throughput drug screening due to intricate in vivo pharmacokinetics and economic limitations. Other cell models, like immortalized human fibroblast lines, have limitations including a singular cell type and lack of structural organization. Additionally, their short viability and dependence on experimenters greatly impede further investigation (Eglen and Reisine 2011). Consequently, the development of antifibrotic drugs targeting heart disease progresses at a slower pace compared to fields like oncology and metabolism due to the absence of appropriate human cardiac fibrosis models.

### Key regulators of cardiac fibrosis

**Fig. 2:**
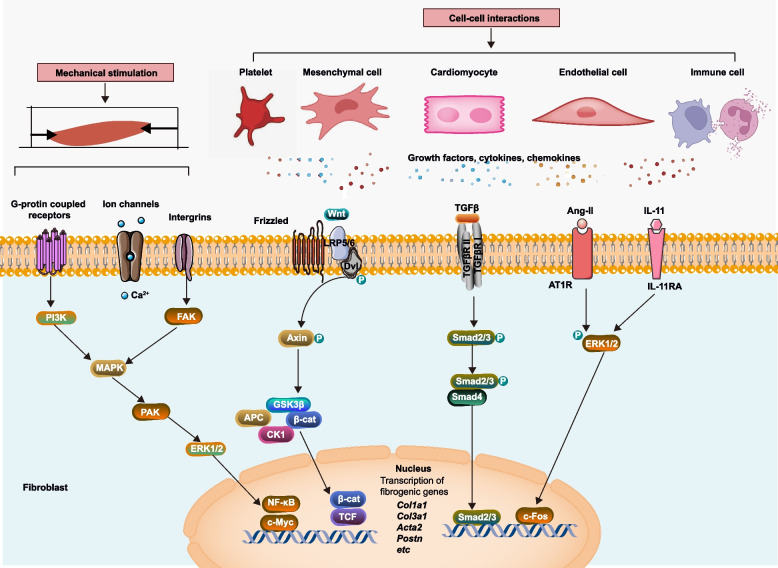
Major signaling pathways involved in cardiac fibrosis. Mechanosensitive pathways play a pivotal role in activating fibroblasts during various cardiac pathological conditions. Integrins, mechanosensitive ion channels, and activation of G-protein coupled receptors all initiate signaling pathways, including FAK, MAPK, and PI-3 K, which mediate the response of fibroblasts to mechanical stress. Secreted signals from diverse cell types have profound effects on phenotypic changes. In response to injury, the heart releases a wide range of cellular factors that trigger and exacerbate the phenotype in a paracrine manner. These secreted factors are implicated in various outcomes such as fibrosis, myofibroblast activation, collagen synthesis, calcification, hypertrophy, and inflammation. TGF-β: transforming growth factor-β; AT1R, type 1 angiotensin II receptor; ERK; extracellular-signal-regulated kinase; FAK, focal adhesion kinase; IL-11RA, IL-11 receptor subunit-α; MAPK, mitogen-activated protein kinase; MR, mineralocorticoid receptor; PI3K, phosphoinositide 3-kinase; TAK1; TGF-beta activated kinase 1, TGFβ-activated kinase 1; TGFβR1, TGFβ receptor type 1. PAK, p21-Activated kinases; NF-κB, The nuclear factor-kappaB; Dvl, Dishevelled; LRP5/6, low-density lipoprotein receptor-related protein; GSK3, glycogen synthase kinase 3; APC, adenomatous polyposis coli; CK1, casein kinase 1; TCF, T cell Factor

Cardiac fibrosis is characterized by the excessive accumulation of extracellular matrix proteins by cardiac fibroblasts and myofibroblasts, serving as a prominent hallmark in various cardiac disorders, including arrhythmia, hypertrophy, and heart failure. This pathological process is triggered by a range of stimuli, such as myocardial injury, inflammatory processes, and mechanical strain. The fibrogenesis cascade is tightly regulated by diverse signaling pathways and various cell types (Fig. 2).

The mechanosensitive activation of fibroblasts may have evolved as a protective mechanism to preserve tissue integrity, mitigating the potentially devastating effects of mechanical forces on tissue structure. However, within the cardiac context, prolonged mechanical tension can lead to sustained fibroblast activation and excessive collagen deposition, ultimately resulting in adverse alterations to myocardial structure and impaired functionality. Mechanical stress can initiate fibroblast activation through various mechanisms, including integrins (MacKenna et al. 1998), mechanosensitive ion channels (Rahaman et al. 2014), and G-protein coupled receptors (GPCRs) (Barnes et al. 2018).

The activation of GPCRs can initiate signaling pathways that coordinate the fibroblast response to mechanical stress. These pathways involve crucial components such as focal adhesion kinase (FAK) (Leask 2013), mitogen-activated protein kinase (MAPK) (Wang et al. 2003), RhoA/Rho kinase (ROCK) (Shimizu and Liao 2016), and phosphoinositide 3-kinase (PI3K) signaling (Frangogiannis 2021). The presence of intracellular calcium ions (Ca^2+^) plays a pivotal role in initiating cardiac fibrogenesis in response to mechanical stress (Lin et al. 2019; Yue et al. 2013; Du et al. 2010). Transient receptor potential (TRP) channels, renowned for their distinctive characteristics, have garnered prominence as the primary ion channels responsible for mediating Ca^2+^ signals in cardiac fibroblasts. There is an increasing body of evidence suggesting the therapeutic potential of numerous TRP channels as targets for drug interventions. Nevertheless, a more comprehensive comprehension of the functions of TRP channels in the heart is indispensable (Feng et al. 2019).

Following myocardial damage, a plethora of signaling molecules such as chemokines, cytokines and growth factors are released, triggering the activation of cardiac fibroblasts through diverse pathways. The RAAS is activated during the process of cardiac fibrosis and interacts with pathways that contribute to fibrosis (Xu et al. 2010). Angiotensin II (Ang II) plays a pivotal role in the transformation of cardiac fibroblasts into myofibroblasts by binding to the Ang II receptor type 1 (AT1R) (Kawano et al. 2000). TGF-β activates fibroblasts through both canonical SMAD2/3 signaling and non-canonical TGF-beta activated kinase 1(TAK1)-mediated p38 phosphorylation (Frangogiannis 2022; Kim et al. 2018; Hu et al. 2018). In summary, the TGF-β signaling pathway assumes a central role in cardiac fibroblast differentiation and the development of cardiac fibrosis (Fig. 2). Furthermore, TGF-β1 signaling induces an upregulation of IL-11 secretion in human fibroblasts, while in vivo studies with global IL-11Ra loss demonstrate reduced interstitial fibrotic remodeling under pressure overload conditions. The impact of IL-11 on fibroblast activation involves post-transcriptional mechanisms mediated by extracellular-signal-regulated kinase (ERK) signaling (Liu et al. 2020).

In mammals, the Wnt signaling pathway plays a crucial role in embryonic development but remains inactive in adult tissues with low turnover, such as the heart (Bastakoty and Young 2016; Aisagbonhi et al. 2011). The Wnt/β-catenin pathway inhibits the destruction complex composed of Axin complex, which includes casein kinase 1 (CK1), adenomatous polyposis coli (APC), and glycogen synthase kinase 3 (GSK3), leading to the accumulation of β-catenin (Frangogiannis 2022). Furthermore, in murine subjects, CFs β-catenin knockout reduces cardiac fibrosis by downregulating collagen type I alpha 1 chain (COL1A1), collagen type III alpha 1 chain (COL3A1), and periostin expression levels (Xiang et al. 2017). Targeting these pathways therapeutically has garnered significant scientific and clinical interest.

### Stem cells provide optimal in vitro models for cardiac fibrosis

Human pluripotent stem cells (hPSCs), including human embryonic stem cells (hESCs) and human induced pluripotent stem cells (hiPSCs), have substantially expanded the availability of human cells for modeling cardiac fibrosis and discovering drugs due to their ability to unlimitedly self-renew and differentiate into types of cells within the body (Parrotta et al. 2020). The in vitro cardiac fibrosis model using hPSCs-derived quiescent cardiac fibroblasts has reported the responsiveness to fibrotic stimulation (Zhang et al. 2019). However, a 2D in vitro system comprising only fibroblasts cannot precisely model disease context of the fibrotic heart, where contractile cardiomyocytes and fibroblasts are closely connected to form the 3D functional tissue.

**Fig. 3:**
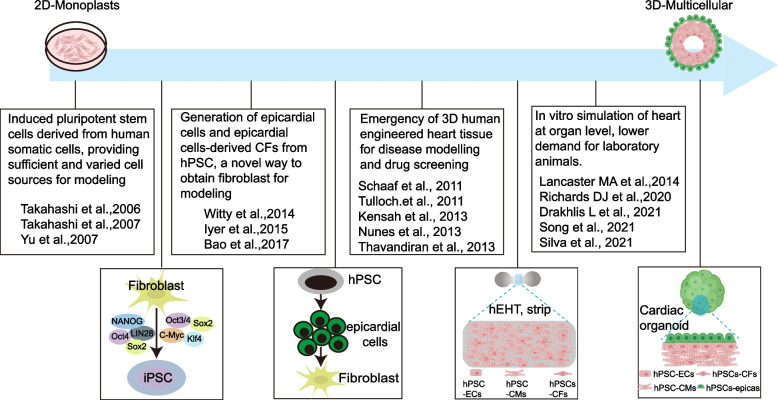
Development of stem cell-based cardiac fibrosis models. The advancement of stem cell technology has facilitated the establishment of models for cardiac fibrosis in the past two decades (Drakhlis et al. 2021; Song et al. 2021; Silva et al. 2021; Richards et al. 2020; Bao et al. 2017; Iyer et al. 2015; Witty et al. 2014; Lancaster and Knoblich 2014; Thavandiran et al. 2013; Nunes et al. 2013; Kensah et al. 2013; Tulloch et al. 2011; Schaaf et al. 2011; Yu et al. 2007; Takahashi et al. 2007; Takahashi and Yamanaka 2006). Due to the self-renewal and pluripotency characteristics of induced pluripotent stem cells, it has become possible to produce target cardiac cells on a large scale in vitro. Building upon this progress, the creation of three-dimensional structures that closely resemble an authentic heart has now become a reality. Oct3/4: organic cation/carnitine transporter 3/4; Sox2: SRY-box transcription factor 2; c-Myc: transcriptional regulator Myc-like; Klf4:KLF transcription factor 4; NANOG: Nanog homeobox; LIN28: Protein lin-28; CFs: cardiac fibroblasts; hPSCs: human pluripotent stem cells; hEHT: human engineered heart tissue; hPSC-ECs: human pluripotent stem cell derived endocardial cells; hPSC-CMs: human pluripotent stem cell derived cardiomyocytes; hPSC-CFs: human pluripotent stem cell derived cardiac fibroblasts

Fortunately, recent advancements in stem cell and tissue engineering technology have significantly facilitated the development of 3D systems at the tissue level. This progress is expected to greatly enhance the construction of more reliable cardiac fibrosis models (Fig. 3). There exist substantial differences in both structure and physiological properties between 2 and 3D cardiac cell cultures (Pontes Soares et al. 2012), which implies that different results can be obtained from either a 2D or a 3D modeling approach. For examples, the cardiac cells grown in a 3D context exhibit smaller size, increased intercellular junctions, and less prominent cytoskeletal network compared to those cultured in a 2D context. Conversely, the cardiomyocytes cultured in a 2D environment demonstrate underdeveloped excitation–contraction coupling, slow action potential conduction, and inefficient energy conversion (Pontes Soares et al. 2012; Abbott 2003). Furthermore, studies have indicated that cells cultured in a 3D setting display reduced sensitivity to drugs and mechanical stimuli as well as decreased resistance to apoptotic signals (Li et al. 2016; Li et al. 2008; Bokhari et al. 2007).

Therefore, employing 3D models offers significant advantages over traditional 2D models for investigating cardiac fibrosis.

The 3D structure closely resembles an actual patient's heart, enabling comprehensive observations of mechanical stimulation on cardiac fibrosis. Various devices have been developed to mimic different forms of force (Occhetta et al. 2018; Kong et al. 2019; Rogers et al. 2019; Bracco Gartner et al. 2023), thereby accelerating researches on the impact of mechanical forces on cardiac fibrosis. Moreover, cardiac cells differentiated from hPSCs cultured in 3D models exhibit a higher level of maturity compared to those in 2D models (Lange et al. 2021) This is attributed to the presence of intercellular interactions, ECM interactions, and microenvironmental stimuli.

Spheroid cultures have been extensively studied in CVDs research for their enhanced integration of biochemical and physiological characteristics, which are similar to real heart within a well-defined 3D architectural microenvironment when compared to 2D monolayer cultures (Beauchamp et al. 2015). The spheroids can be derived from various cell types, including both somatic and stem/progenitor cells, as well as resident cardiac stromal cells. Despite their simplicity, these spheroids offer valuable preliminary models for investigating complex pathological conditions such as tissue stiffening and fibrosis (Edmondson et al. 2014; Polonchuk et al. 2017; Sacchi et al. 2020; Garoffolo et al. 2022). Cardiac organoid represents a more complex form of 3D microtissue, which are self-assembling structures of cardiac cell types obtained from the proliferation and differentiation of hPSCs. The cardiac organoids are attempted to replicate cell–cell interaction, cell-ECM interaction, and organ architecture and function at a microscale level, as proximal as possible to the in vivo histological features (Nugraha et al. 2019; Lewis-Israeli et al. 2021). More significantly, cardiac organoids serve as a crucial model for the discovery and evaluation of novel drugs and treatments, transforming "personalized medicine" from bench to bed. The self-organizing nature of cardiac organoids enables considerably higher throughput compared to engineered heart tissues (EHTs) or animal models in drug screening owing to their simplified fabrication process and smaller cell count per organoid (~ 5,000 cells) (Drakhlis et al. 2021). In addition, organoids enable personalized medicine approaches using the hiPSCs with patient’s genetic background and to evaluate drug effects on human cardiac development and gene expression pattern. Based on these advantages, cardiac organoids are already used in drug testing for CVDs (Lee et al. 2020; Paik et al. 2020). Mi-Ok Lee et al. established an in vitro 3D microtissues derived from hESCs to model cardiac fibrosis, they demonstrated that the addition of appropriate amount of CD44^+^ human mesenchymal stem cells (hMSCs) (about 40%) derived from hESCs better mimics the pathological process of cardiac fibrosis (Lee et al. 2019). Similarly, the cardiac fibrosis model constructed by Iseoka et al. demonstrated that cardiac tissues comprising 50%–70% cardiomyocytes exhibited enhanced responsiveness to fibrotic stimulation, thereby enabling precise screening of anti-fibrotic drugs (Iseoka et al. 2021).

Despite the rapid advancements in this field, there is still a lack of highly efficient and reproducible methods for the generation of cardiac organoids and universally applicable culture conditions for all cardiac cell types. The generation of cardiac organoids has not yet undergone the sequential processes of looping, ballooning, trabeculation, and compaction, which are crucial for chamber formation in a native heart (Kim et al. 2021). Moreover, current cardiac organoids lack spatial organization and perfusable blood vessels, limiting their size and posing challenges for long-term maintenance as they grow larger. Additionally, the pumping function mediated through muscle contraction and vascular transportation has not been observed thus far (Zhao et al. 2019; Zhang et al. 2021; Feng et al. 2022). Therefore, cardiac organoids derived from hPSCs exhibit characteristics more reminiscent of fetal hearts rather than adult hearts.

Although current cardiac organoids are unlikely to fully replace animal models in preclinical studies, ongoing efforts will help bridge the gap between in vivo and in vitro applications in the future (Kim et al. 2022; Sahara 2023).

### Stem cell therapy for treating cardiac fibrosis

In recent years, stem cell technology has provided a ray of hope for the treatment of numerous ‘intractable diseases’, and its remarkable efficacy in combating heart diseases has equally demonstrated the immense potential of stem cells in addressing cardiac conditions. Given that cardiomyocytes are terminally differentiated, any damage incurred results in permanent loss and subsequent replacement by scar tissue within the damaged myocardium. This process often ends with complications such as heart failure and malignant arrhythmias, significantly impacting overall quality of life. Researchers have harnessed the regenerative potential of different types of stem cells, such as hMSCs, hESCs and hiPSCs, to target the damaged heart tissue and promote its repair and regeneration. One of the primary mechanisms by which stem cells combat cardiac fibrosis is their capacity to differentiate into specialized cardiac cells. These cellular components play crucial roles in the regeneration of damaged myocardial tissue and restoration of cardiac function (Zhao et al. 2022). The secretion of various growth factors and cytokines by stem cells further facilitates the recruitment of endogenous repairing cells and stimulates the formation of new blood vessels, thereby promoting the hemodynamics of the heart (Ishigami et al. 2018; Quijada and Sussman 2014).

MSCs possess a range of characteristics, including anti-fibrotic, anti-inflammatory, anti-apoptotic, immune-modulatory, and pro-angiogenic properties through secreting various molecules with anti-inflammatory and immune-modulatory activities, thereby promoting the regeneration of damaged heart tissues (Razeghian-Jahromi et al. 2021). Extensive preclinical and clinical investigations have demonstrated the potential of MSCs transplantation in offering protection against diverse CVDs such as acute myocardial infarction (MI), both ischemic and non-ischemic heart failure, chemotherapy-induced cardiomyopathy, and myocarditis. Notably, these reports and clinical trials over the past decades have indicated limited cardiomyogenic potential and modest improvement in cardiac function for ischemic cardiomyopathy of MSCs based therapy (Silva et al. 2005; Hare et al. 2012; Mathiasen et al. 2015).

hPSCs possess clonogenic, self-renewing, and pluripotent properties, making them highly expandable and capable of in vitro differentiation into cardiomyocytes (hPSC-CMs). This feature illustrates the potential to obtain abundant cardiomyocytes for transplantation  (Chen et al. 2023). Thus, hPSCs also serve as promising resources for the treatment of myocardial fibrosis **(**Fig. 1**)**. The transplantation of hPSC-CMs holds the potential to directly enhance cardiac function in individuals with reduced fibrosis and increased vascular density. Additionally, hPSC-CMs can enhance cardiac tissue regeneration and repair processes by secreting growth factors, cytokines, and other signaling molecules (Dessouki et al. 2020; Wu et al. 2020). However, the limited engraftment rate of transplanted cells remains a significant hindrance to the effectiveness of this cell therapy (Ishigami et al. 2018; Qu et al. 1998; Tang et al. 2010). Safety concerns such as arrhythmias and potential tumorigenesis have been reported in hPSC-CMs-based therapy (Chong et al. 2014).

The ideal approach for hPSC-CMs transplantation has been extensively investigated through numerous studies, encompassing three distinct methodologies: coronary artery injection, myocardial injection of cell sheets, and utilization of 3D patches. However, the direct injection of dissociated single cells into the myocardium or coronary artery yields a transplantation success rate below 10% (Hsiao et al. 2013; Behfar et al. 2014). Therefore, several studies have been dedicated to the development of innovative injection techniques aimed at raising cell retention rates, such as co-transplantation with human MSCs that release anti-apoptotic factors (Templin et al. 2012). In addition, recent years have witnessed the development of various novel tissue engineering strategies aimed at enhancing cell transport in myocardial regeneration therapy. Engineered cell sheets, as compared to direct injection, owe the advantage of delivering a large number of cells to damaged tissues without engendering transplanted cell loss or causing damage to the host myocardium. Moreover, the application of hPSC-CMs embedded in 3D patches promotes their continuous maturation and might provide further value as a potential therapy (Zhang et al. 2013; Sun and Nunes 2017; Gao et al. 2018).

Recently, researchers have been investigating multiple approaches to enhance the engraftment of hPSC-CMs. Notably, the Ye laboratory has reported the promotion of thymosin β4 (Tβ4) in the implantation of hPSC-CMs in a subacute myocardial infarction pig model (Tan et al. 2021). They have confirmed that combination therapy with Tβ4 can significantly enhance hPSC-CMs implantation and angiogenesis, promote the proliferation of endogenous cardiomyocytes and endothelial cells and alleviate adverse cardiac remodeling. Importantly, no safety concerns such as ventricular arrhythmias or tumor formation were observed. Zhao et al. also demonstrated that over-expression of Cyclin D2 in hPSC-CMs enhances heart function, reduces fibrotic scar size and ventricular hypertrophy, and decreases cardiomyocyte apoptosis with increased vascular density (Zhao et al. 2021).

Considering that cardiac fibrosis commonly accompanies the majority of CVDs, the clinical application of hPSC-CMs holds great promise in treating these diseases. The immense potential of stem cell-based therapy for CVDs ensures that both preclinical and clinical investigations will continue unabated. At present, there are four ongoing clinical trials utilizing hPSC-CMs to treat patients with CVDs (Sridharan et al. 2023).

### Cell reprogramming technique for treating cardiac fibrosis

**Fig. 4:**
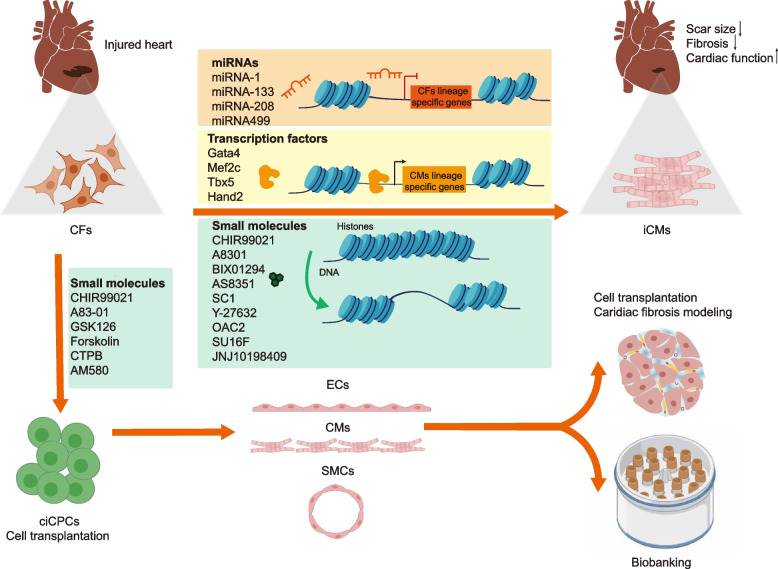
Cell reprogramming technique for cardiac fibrosis therapy. Cardiac fibroblasts could be directly reprogrammed to cardiomyocyte-like cells through microRNAs, transcription factors and small molecules in vivo to achieve in situ cardiac tissue repair. The microRNAs and transcription factors switch the cell fate through limiting the original lineage specific genes expression or promoting the target lineage specific genes expression, and the small molecules function by promoting the chromatin opening at lineage specific loci. Cardiac fibroblasts could also be indirectly reprogrammed to cardiac progenitor cells through small molecules in vitro*,* which can be used for transplantation or differentiate into various cardiac cell types in a large scale for cell transplantation, disease modeling, and biobanking of patient-specific samples. CFs, cardiac fibroblasts; miRNAs, microRNAs; iCMs, induced cardiomyocyte-like cells; ciCPCs, chemically induced cardiovascular progenitor cells; ECs, endothelia cells; CMs, cardiomyocytes; SMCs, smooth muscle cells

Given that direct reprogramming can generate reprogrammed cells in situ in diseased organs of animal models, its utilization may overcome technical challenges associated with iPSC technology, such as in vitro reprogramming and large-scale amplification. Cardiac fibroblasts have been the primary source for cardiomyocyte conversion due to their activation and demonstrated contribution to fibrosis and scar formation following heart injury. The in vivo reprogramming of cardiac fibroblasts has resulted in the replenishment of cardiomyocyte pools and reduced scar formation, suggesting a potential pathway for treating cardiac fibrosis. Research has indicated that by inducing trans-differentiation of fibroblasts into various cell types, including cardiomyocytes (Cao et al. 2016) or endothelial cells (Han et al. 2021; Lee et al. 2017), it is possible to mitigate the accumulation of fibrous tissue and facilitate tissue repair (Fig. 4).

This trans-differentiation process can be achieved through various methods, including the utilization of transcription factors (Zhang et al. 2017), gene editing tools (Lee et al. 2017), or small molecules (Cao et al. 2016). The first approach introduced involves coaxing fibroblasts to undergo in vitro reprogramming into cardiomyocyte-like cells, either by redirecting incompletely reprogrammed cells towards a cardiac phenotype using the inherent reprogramming strategy or by introducing the three transcription factors GATA binding protein 4 (GATA4), myocyte enhancer factor 2C (MEF2C), and T-box transcription factor 5 (TBX5) (GMT) into cultured fibroblasts (Efe et al. 2011; Ieda et al. 2010). Subsequently, efforts were made to investigate the acquisition of reprogrammed and authentic cardiomyocytes in vivo. Jayawardena et al. initially demonstrated successful conversion of cardiac fibroblasts into cardiomyocytes within scarred and peri-infarct areas of mouse hearts through direct lentiviral delivery of miRNAs (miRNAs-1, 133, 208, and 499), with enhanced reprogramming effects observed when combined with Janus kinase (JAK) inhibitor I treatment (Jayawardena et al. 2012). In a study leaded by Dr. Qian, the researchers discovered that fibroblasts undergoing proliferation were directly reprogrammed into relatively mature cardiomyocytes with authentic action potentials (APs) and contractile ability through over-expression of GMT mediated by a retroviral system. This was confirmed by strict lineage tracing experiments. Additionally, they reported an enhanced reprogramming efficiency of 12% compared to the typical 5–10% in vitro efficiency. The reprogrammed cardiomyocytes exhibited similar transcriptional profiles and physiological features as endogenous adult cardiomyocytes, suggesting the influence of both cellular and extracellular environments. Importantly, following the delivery of GMT for 8–12 weeks in post-MI mice, new cardiomyocytes were observed in scar areas, aligning with significant and long-lasting improvements in cardiac function and reduced scar size as demonstrated by echocardiography and Magnetic Resonance Imaging (MRI) (Qian et al. 2012).

Given the presence of species variations, more intricate reprogramming cocktails were required for human cardiac reprogramming. Fu et al. employed GMT in conjunction with estrogen-related receptor gamma (ESSRG), mesoderm posterior bHLH transcription factor 1 (MESP1), myocardin (MYOCD), and zinc finger protein, multitype 2 (ZEPM2) to convert human fibroblasts into human induced cardiomyocyte-like cells (hiCMs) (Fu et al. 2013), while Wada et al. demonstrated that a combination of GMT along with MESP1 and MYOCD was sufficient for hiCM conversion in coculture with murine cardiomyocytes (Wada et al. 2013). In recent years, Zhou et al. developed an optimized protocol using MGT plus miR133 based on single-cell transcriptomic analysis, which achieved an efficiency of 40%-60% in generating hiCMs (Garbutt et al. 2020; Zhou et al. 2019).

Although new reprogramming protocols have been investigated to enhance the efficiency of reprogramming, cardiac cells generated through genetic methods exhibit heterogeneity and a low proportion of truly functional hiCMs that spontaneously beat and display cardiac Aps (Xie et al. 2022). In comparison to viral-based gene delivery methods, small molecules offer the advantage of being non-immunogenic and cost-effective, along with easily standardized protocols. Our research group has successfully reprogrammed human fibroblasts into functional cardiomyocytes (chemically induced cardiomyocytes, ciCMs) using a combination of nine small molecules: CHIR99021, A83-01, BIX01294, AS8351, SC1, Y-27632, OAC2, together with two inhibitors of platelet-derived growth factor receptors, namely SU16F and JNJ10198409 (Cao et al. 2016). The underlying mechanism is that 9C can induce an epigenetic state characterized by open chromatin structure in somatic cells, enabling them to respond to external cardiogenic signals (Fig. 4). Moreover, the transplantation of 9C-treated human foreskin fibroblasts into the infarcted hearts of immunodeficient mice resulted in robust expression of cardiac markers, well-organized sarcomeres, and partial re-muscularization within the infarcted area. The ciCMs closely resembled human cardiomyocytes in terms of transcriptome, epigenetic features, and electrophysiological properties. This discovery establishes a foundation for potential in situ repair of the heart through targeted modulation of endogenous cardiac fibroblasts using small molecules.

Using similar strategy, we have recently reported the generation of chemically induced cardiovascular progenitor cells (ciCPCs) from mouse and human fibroblasts, which possess multipotency to differentiate into various types of cardiovascular cells using a transgene-free reprogramming approach involving six small molecules: CHIR99021, A83-01, GSK126, Forskolin (an adenylyl cyclase activator), CTPB (a P300 histone acetyltransferase activator), and AM580 (a RARα activator) (Wang et al. 2022). Subsequent transplantation of these ciCPCs into infarcted mouse hearts resulted in improved animal survival and cardiac function for up to 13 weeks post-infarction. Furthermore, considering the autologous nature of ciCPCs, these infinitely renewable cardiovascular cells offer the potential for biobanking patient-specific stem cells, thereby playing a crucial role in personalized cell therapy and precise drug screening targeting cardiac fibrosis.

Despite the considerable potential of stem cell therapy in addressing cardiac fibrosis, there are still several obstacles need to be overcome. These challenges encompass the selection of appropriate stem cells sources, concerns regarding biosafety during cell transplantation, and the necessity to improve cell survival and retention rates, among other factors. The advantages and disadvantages of stem cell transplantation and cell reprogramming for cardiac fibrosis therapy are summarized in Table 1. Moreover, in order to develop more effective and targeted treatment approaches, it is crucial to gain a comprehensive understanding of the intricate pathological mechanisms involved in fibrosis.

**Table 1:** The advantages and disadvantages in stem cell transplantation and in vivo cell reprograming for cardiac fibrosis therapy

Therapy pathway	Advantages	Disadvantages	References
Stem cell transplantation	1). Diverse sources of stem cells 2). Precision therapy of injury heart 3). Exhibits a dose-dependent function 4). Induce both muscularization and vascularization in the injured heart	1). Immune rejection 2). Biosafety concerns 3). Adverse cardiac effects (e.g., arrhythmias) 4). Low immaturity of transplanted cardiac myocytes 5). Low retention rate of transplanted cells	Lou et al. 2023 Querdel et al. 2021 Gao et al. 2018 Liu et al. 2018
In vivo Cell reprograming	1). In situ repair of the injured heart 2). Cardiac fibroblast-specific 3). Reduce cardiac fibrosis and generate new cardiomyocytes in the same time	1). Low efficiency and high cost 2). Biosafety concerns 3). Technical complexity 4). Emerging mutability	Tang et al. 2022 Garry et al. 2021 Muraoka et al. 2019 Miyamoto et al. 2018

## Conclusions and perspectives

In summary, stem cell therapy for cardiac fibrosis is an advancing field with promising prospects for the future. Ongoing research and technological advancements are propelling these therapeutic approaches towards potential success. However, several challenges persist in current stem cell treatments. Further investigation is required to address issues such as the selection of appropriate stem cell sources, biosafety concerns, and the feasibility to monitor treatment efficacy. Moreover, ensuring the successful engraftment and survival of transplanted stem cells into the heart remains to be tackled. Many transplanted cells do not exhibit long-term viability or fail to differentiate into functional cardiac cells, thereby impeding the overall effectiveness of this therapy. To ensure the safety and efficient implementation of these stem cell-based approaches, rigorous clinical studies and repeated validation are indispensable. Efforts are underway to establish a high-throughput drug screening platform for the development of novel therapies. Striking a balance between complexity and user-friendliness is pivotal for this platform. Through comprehensive research and validation, we can surmount these challenges and unlock the full potential of stem cell therapy for cardiac fibrosis in the future.

## Data Availability

Not applicable.

## Abbreviations

ACE: Angiotensin-converting-enzyme
α-SMA: α-Smooth muscle actin
APC: Adenomatous polyposis coli
APs: Action potentials
Ang II: Angiotensin II
AT1R: Ang II receptor type 1
ciCPCs: Chemically induced cardiovascular progenitor cells
ciCMs: Chemically induced cardiomyocytes cells
CMs: Cardiomyocytes
CFs: Cardiac fibroblasts
CVDs: Cardiovascular diseases
COL1A1: Collagen type I alpha 1 chain
COL3A1: Collagen type III alpha 1 chain
CK1: Casein kinase 1
ECM: Extracellular matrix
EHTs: Engineered heart tissues
ESSRG: Estrogen-related receptor gamma
FAK: Focal adhesion kinase
GPCR: G-protein coupled receptors
GATA4: GATA binding protein 4
GSK3: Glycogen synthase kinase 3
hESCs: Human embryonic stem cells
hESC-CMs: Human embryonic stem cell-derived cardiomyocyte
hiPSCs: Human induced pluripotent stem cells
hiPSC-CMs: HiPSC-derived cardiomyocytes
hPSCs: Human pluripotent stem cells
hPSC-CMs: HPSC-derived cardiomyocytes
hPSC-CFs: HPSC-derived cardiac fibroblasts
hPSC-ECs: HPSC-derive endothelial cells
hMSCs: Human mesenchymal stem cells
hiCMs: Human induced cardiomyocytes-like cells
IL: Interleukin
IPF: Idiopathic pulmonary fibrosis
MI: Myocardial infarction
MAPK: Mitogen-activated protein kinase
MEF2C: Myocyte enhancer factor 2C
MRI: Magnetic Resonance Imaging
MYOCD: Myocardin
MSCs: Mesenchymal stem cells
NASH: non-alcoholic steatohepatitis
PDGF: Platelet-derived growth factor
PI3K: Phosphoinositide 3-kinase
RAAS: Renin angiotensin aldosterone system
ROCK: RhoA/Rho kinase
TGF-β: transforming growth factor-β
Tβ4: Thymosin β4
TRP: Transient receptor potential
TBX5: T-box transcription factor 5
Wnt/β-catenin: Wnt family/β-catenin
ZFPM2: Zinc finger protein, multitype 2
